# Worker Personality and Its Association with Spatially Structured Division of Labor

**DOI:** 10.1371/journal.pone.0079616

**Published:** 2014-01-30

**Authors:** Tobias Pamminger, Susanne Foitzik, Katharina C. Kaufmann, Natalie Schützler, Florian Menzel

**Affiliations:** Department of Evolutionary Biology, Institute of Zoology, Johannes Gutenberg University of Mainz, Mainz, Germany; Universidade de São Paulo, Faculdade de Filosofia Ciências e Letras de Ribeirão Preto, Brazil

## Abstract

Division of labor is a defining characteristic of social insects and fundamental to their ecological success. Many of the numerous tasks essential for the survival of the colony must be performed at a specific location. Consequently, spatial organization is an integral aspect of division of labor. The mechanisms organizing the spatial distribution of workers, separating inside and outside workers without central control, is an essential, but so far neglected aspect of division of labor. In this study, we investigate the behavioral mechanisms governing the spatial distribution of individual workers and its physiological underpinning in the ant *Myrmica rubra.* By investigating worker personalities we uncover position-associated behavioral syndromes. This context-independent and temporally stable set of correlated behaviors (positive association between movements and attraction towards light) could promote the basic separation between inside (brood tenders) and outside workers (foragers). These position-associated behavior syndromes are coupled with a high probability to perform tasks, located at the defined position, and a characteristic cuticular hydrocarbon profile. We discuss the potentially physiological causes for the observed behavioral syndromes and highlight how the study of animal personalities can provide new insights for the study of division of labor and self-organized processes in general.

## Introduction

Division of labor is a characteristic trait of many human societies and one factor responsible for their dominant position on a global scale. By separating complex tasks into simpler subtasks, which are performed by specialists, a social group can achieve an overall increase in productivity [1]. However, humans are not the first species who developed division of labor. Millions of years before the neolithic revolution, social insects started to allocate tasks among different members of the group. In societies of ants, wasps, bees and termites, reproduction as well as other tasks (e.g. garbage disposal, brood care and guarding the entrance) are carried out by specialized individuals [2].

In social insect societies, the distribution of tasks between different group members is controlled by various parameters including age, experience, physiology and morphology [3]–[7]. As a consequence, the probability to perform a specific task varies among the individual members of a colony, at a given point in time, resulting in division of labor. The probability of an individual to perform a specific task is termed ‘task threshold’ and was shown to be an essential aspect in the evolution of division of labor [8]. Recent work suggests that, besides threshold variation, the spatial position of an individual is a fundamental, but so far neglected aspect for generating division of labor [9]–[12]. Its importance stems from the fact that many essential tasks can only be performed at specific locations, and cues that indicate the demand for a specific task is only available to individuals in the direct vicinity [13]. For example, larvae can be cared for only inside the brood chambers (or the nest), and food resources can only be detected and collected outside. Consequently, division of labor requires not only individuals with a high probability to perform an essential task, but in addition these individuals have to be in the right spatial position to respond in time.

Besides the general problem of organizing spatially structured division of labor, insect colonies face the challenge of temporal fluctuations in the workforce demand for specific tasks (e.g. seasonal variation in brood number and associated tasks). In some ant species, the problem of worker allocation between tasks is solved by worker switching from their current task to the task in high demand. They have been shown to utilize caste-specific cuticular hydrocarbon profiles to fast and accurately estimate the number of individuals that perform a certain task and use this information to asses task demand make an informed decision [14].

Complex patterns such as division of labor, and regulation of such, were suggested to emerge by self-organization [15]. Self-organization is defined as spontaneous formation of complex pattern as a result of the involved agents following simple and local rules only. If individual workers followed intrinsic behavioral rules which determine their spatial position, and if such rules were coupled with a low threshold of performing a task specific to this location (e.g. brood care inside the nest), then division of labor could be achieved via self-organization. Such correlated sets of behavioral traits have been termed ‘animal personalities’ or ‘behavioral syndromes‘ [16]. On a larger scale, animal personality can predict the migratory probability of individuals and consequently their spatial position over a broad geographic scale [17]. The same principle, on a much smaller scale, might be applied to the spatial position of social insects. The behavioral mechanisms underlying spatially structured division of labor and its potential physiological underpinning will be the topic of this paper. Using the ant *Myrmica rubra* as a study organism, we will investigate four aspects of spatially structured division of labor:

1. **Spatial fidelity.** In accordance with recent publications we hypothesize the different behavior castes will exhibit spatial fidelity.
2. **Spatial flexibility.** We hypothesize that an ant will be able to detect the lack of workforce performing complementary task and will compensate by task switching.
3. **Personality.** We hypothesized that if ants exhibit temporally stable behavior concerning activity and phototaxis, outside workers will be characterized by increased positive phototactic behavior and higher activity. The other behavioral experiments were performed in order to get a more complete picture focusing in task-specific thresholds like interaction with cricket legs.
4. **Morphology, physiology, and chemical cues.** We hypothesize that behavioral casteswillshow caste-specific differences in their physiology (morphology, reproductive status and cuticular hydrocarbon profile).

## Materials and Methods

### Colony Collection and Housing

We collected 16 colonies of *M. rubra* in September 2011 and additional 6 colonies in spring 2012 at the Ober-Olmer Wald (49° 57.752′ N, 8° 11.184′ E) near Mainz, Germany. *M. rubra* is native to Germany and is neither endangered nor protected under German law. The collection permit was issued by Forstamt Rheinhessen (Forstrevier Ober-Olm): Genehmigung Oberolmer Wald. The ant colonies were censused and allowed to move into nest boxes with plastered floor (17.5×23.5×10 cm) with a nest cavity imprinted in the floor and covered with red foil. Ants were kept at room temperature during the experiments and fed crickets and honey three times a week. Water was supplied *ad libitum*. All colonies contained at least four queens (macrogyne) and a minimum of 400 an a maximum of 1300 workers at the time of collection.

### 1. Spatial Fidelity

We collected workers found in four spatial positions potentially associated with a specific task: 1. within the nest directly on the brood (caring for the brood, hereafter **B**) 2. Within 1 cm of a queen (tending the queen, **Q**) 3. Within 1 cm of the nest entrance (guarding, **E**) and 4. Outside the nest (scouting or foraging, **O**). From 11 colonies, 10 individuals per position and colony were individually marked using Edding® (edding, Germany) varnish (*N = *440). Every individual was observed approximately 10 times within 21 consecutive days. At each scan, the position of the individual was recorded (near queen; near brood; at entrance; and outside). If the position was ambiguous, such as within 1 cm of both brood and queen, we recorded both, but assigned the closer item to the individual. If individuals died during the experiment, a replacement worker from the same position was marked and observed for the remaining time of the experiment. Data of these replacement individuals were only included in the analysis when their behavior was scanned at least five times.

We calculated the proportions of scans in which each worker had been recorded at each of the four positions. These proportions were arcsin(sqrt)-transformed and entered as a response variable into a linear mixed-effects models with worker position as fixed and colony as random factor. These calculations were performed using R 2.15.1 [18]. As a *post hoc* test, we used the individual factor levels computed by the model using the *lme* command in the R package *nlme* [19].

### 2. Spatial Flexibility

Based on the observational data of the previous experiment, three artificial subnests per colony were created. Each sub-nest contained a randomly chosen queen, 10 brood items and 10 workers, which were found in the same position (either brood, queen or outside) in at least 50% of all the observations. We were unable to set up sub-nests containing only entrance workers because none of the colonies contained enough workers satisfying our criteria. These subnests were transferred into three-chambered plastic nest boxes (9.5×9.5×3 cm) with a plastered floor. Thus, they were substantially smaller than the nests used for the previous experiment. Over a period of 14 days, every colony was scanned 20 times and the position (brood, queen, entrance, outside) of every worker was recorded.

The statistical analysis was based on the three different positions compared to the first experiment (inside within 2 mm of brood, inside at least 2 mm from brood, outside), as we never found ants directly in the nest entrance. We used the same statistical procedure as in the previous analysis.

### 3. Personality

#### Worker selection

We randomly selected four *M. rubra* colonies and marked workers detected in three spatial positions (brood, entrance and outside). Two days after marking them, their position was checked again. All individuals who were found at the same position as previously, were now individually marked. In total, we selected six workers per colony and spatial position (N = 24) except for ‘entrance’, where we only encountered five workers in two of the four colonies (N = 22). All workers were tested in seven behavioral experiments, which were conducted in a random order. After each trial the arena was cleaned with 70% ethanol.

#### 3a. Phototaxis

This experiment consisted of two assays both conducted in a plastic petri dish (Ø 10 cm). One half of the dish was darkened and separated from the other half by a cardboard wall with a small opening (0.5×0.5 cm) in the middle, thus creating a dark and light half of the arena. Each ant was tested twice in this arena, and put into the center of the light and the dark half once each. The order of the two assays was randomized. We recorded the time the ant spent in the light half during 120 s, and averaged these values over the two assays.

#### 3b. Exploration

The ant was placed into a large plastic arena (36×30 cm) with a grid of 4×4 cm squares on the floor. After 20 s of acclimatization, the ant was followed for 120 s and the number of novel grid squares entered during this time was recorded. This experiment gave a measure of the exploration tendencies of the tested individuals.

Experiments 3c–3g were carried out in small petri dishes (Ø 3.5 cm), and the ant and the object tested were placed into its center. After 20 s acclimatization, the time the ant spent interacting with the object was recorded over the next 120 s.

#### 3c. General activity

In this experiment, no object was placed in the arena, and we recorded the time the ant spent moving around in the arena. This assay was conducted to give a base measurement of the activity level of the ant.

#### 3d. Response to dead non-nestmate

The ant encountered a dead non-nestmate which was killed three days earlier, but frozen until the start of the experiment. The time the ant spent in direct contact (antennating, grooming or carrying) was recorded. This assay measured a worker’s reaction to an potential intruder (i.e. its ‘curiosity’). At the same time the alien ant could elicit defense behavior. This assay was to indicate curiosity to a novel object interesting to workers and potential recondition of intruders but eliminated the behavioral variation induced by the opponent, as freshly frozen ants are not identified as dead by ant workers [20]–[22].

#### 3e. Foraging behavior

In this experiment, a cricket leg was placed in the center of the arena. The ant was observed for 120 s and the time the ant spent interacting (both antenna in contact with the leg) with it was recorded. As we routinely fed our ants with crickets, this test was to indicate the ant’s inclination to forage protein.

#### 3f. Brood care

The ant was confronted with a randomly chosen larva of their own colony. During 120 s, we recorded the time it interacted with the larva (i.e. grooming, feeding, antennating, or carrying).

#### 3g. Aggression

The ant was carefully touched on the head with the tip of a cotton swab. We recorded its reaction, and defined four different categories: (1) flight, (2) mandible spreading, (3) biting, and (4) stinging. These behaviors reflect increasing levels of aggression.

#### Statistical analysis

In order to detect differences in the behavior among ants from the three different positions (brood, entrance and outside), we used principal component analysis (PCA) on all recorded behaviors using R 2.15.1 [18]. Each principal component with an eigenvalue >1 was entered as a response variable into a separate PERMANOVA [23] with position as a fixed and colony as a random factor (all interactions were permitted). As a *post hoc* test we calculated a pairwise comparison between all groups using the same PERMANOVA settings. We used the software PRIMER 6 ver. 6.1.14 (including the PERMANOVA+ add-in, version 1.0.4; PRIMER-E Ltd), and performed all PERMANOVAs with 9999 permutations using euclidian distance as a distance estimate. In case of a low number of unique permutations (n<1000) we used a Monte Carlo procedure to generate the p values following the suggestions of software manual [23]. We used the same procedure for all consecutive PERMANOVAs if not noted otherwise. We used MDS (multi-dimensional scaling) to visualize the worker personalities.

#### 3.1 Consistency of personality traits

To test for the consistency of the behavior of individual workers, we focused on three behavioral assays: phototactic, activity and brood care. We chose four colonies in early 2012 and selected brood-caring and outside workers following the selection procedure described above. After marking them individually, all ants were tested twice in all three behavioral essays with 10 days in between trials. In the following autumn we collected two additional colonies and repeated the behavioral essays described above. In total we gathered data from 65 individuals belonging six different colonies.

To test for behavioral consistency we ran a PCA over all recorded behaviors separately for both test days (PCA1 and PCA2). We inspected the resulting PC axes of both PCAs for consistency regarding loadings, eigenvalue and per cent explained variation. After discovering almost identical factor loadings and Eigenvalues for both pairs of PCs (Table S2 and S3 in file S1) we entered the first PC1 (of PCA1) as a response variable in an ANCOVA analysis with PC1 (of PCA2) and colony origin as predictors to account for the non independence of the individual worker data points The same procedure was applied for PC2 (of PCA1 and PCA2). The calculation were performed using R 2.13.1 [18].

### 4. Physiology

#### 4.1 Body allometry

To test for morphological differentiations, we measured workers from four different colonies found at four spatial positions: within 1 cm of brood, within 1 cm of queen, within 1 cm of the nest entrance, and outside the nest. Four morphological measurements (head width, head length, thorax width and thorax length) were taken using a Leica stereomicroscope (S8AP0, Wetzlar, Germany) and the Leica Application Suite software (Version 3.8).

For the morphological analyses we measured head length, head width, thorax length, pronotum width, of 12–18 workers of ten colonies (N = 131). Each of the four positions (queen, brood, entrance and outside) was represented by 20–40 workers in total, or 0–7 workers per colony (median: 3). A principal components analysis (PCA) was performed over head length, head width, thorax length and pronotum width, using R 2.15.1 [18]). All principal axes with eigenvalues >1 were further analyzed.

#### 4.2 Reproductive status (ovary activation)

The ovaries of all workers participating in the individual behavioral tests were dissected after the experiments and their longest and shortest ovariole were measured using the microscope and settings described above. Using ovary length is a good indicator for the activation status of a worker's ovaries because it is often the first step before egg formation starts. For the statistical analysis the average ovary length of the individual workers was entered as a response variable in the PERMANOVA analysis.

#### 4.3 Cuticular hydrocarbon profiles

In 16 colonies, the cuticular hydrocarbons of single workers from four nest positions (brood, queen, entrance, outside; n = 2 per colony and position) were extracted. For details on the extraction and GC-MS settings see ESM. The software MSD ChemStation (Version E.02.02) for Windows was used for data acquisition. We only considered substances that were present in ≥20% of the samples and had an average abundance of ≥0.5% in at least one of the four nest positions. The hydrocarbons were identified by FM based on retention indices [24] and diagnostic ions.

The relative cuticular hydrocarbon quantities were transformed according to [25]. Differences between positions (brood, queen, entrance and outside) and colonies were analyzed using a PERMANOVA using Bray-Curtis as distances estimate. To test for qualitative differences in cuticular hydrocarbon profiles between worker groups, we grouped the cuticular hydrocarbons in four substance classes (*n*-alkanes, *n*-alkenes, mono-, di- and tri-methylalkanes), and used linear mixed-effects models with worker origin as fixed and colony as random factor (*lme* command in the R package *nlme,* [19]) on the arcsin(sqrt)-transformed proportion of the four substance classes as response variables.

## Results

### 1. Spatial Fidelity

**Figure 1:**
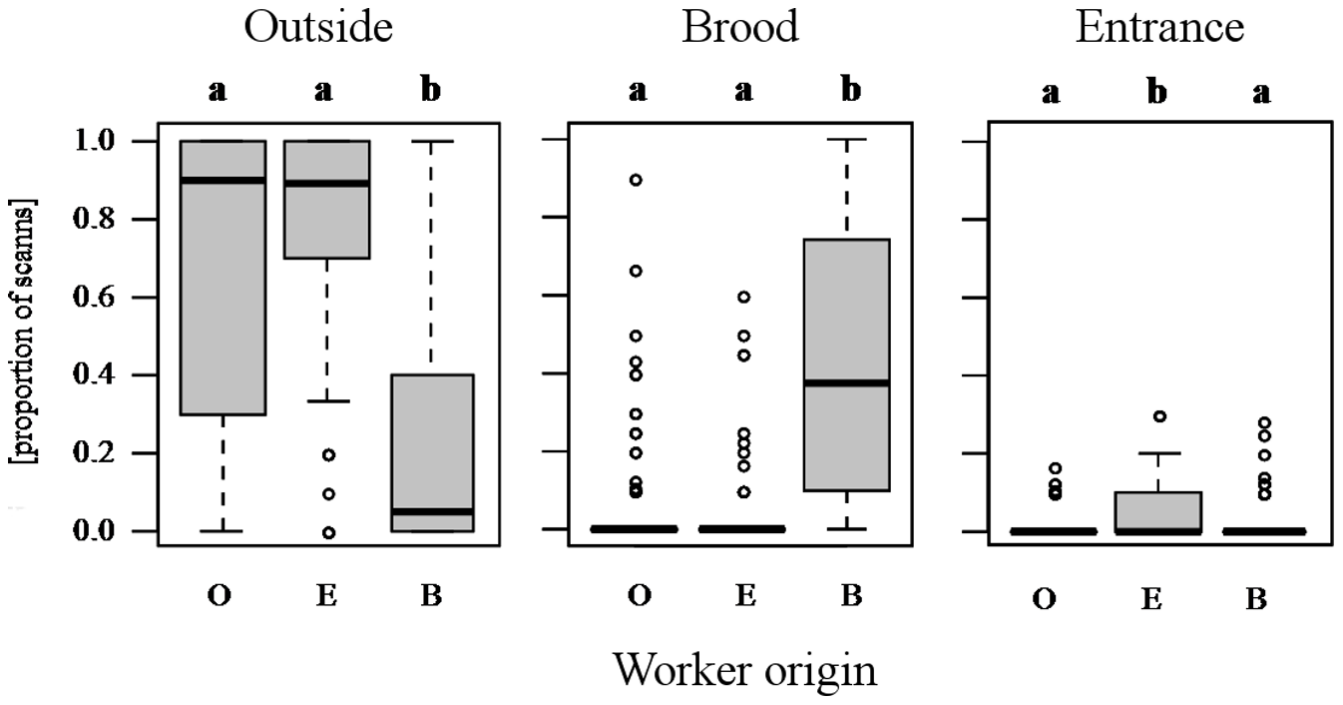
Spatial fidelity of *M. rubra* workers within colonies. Presented is the proportion of scans a worker of a given origin (O = outside, E = entrance, B = brood and Q = queen) was detected at the one of the four monitored locations during experiment 1. Presented are medians and quartiles, circles indicate outliers. Significant differences between the positions (p<0.0001) are indicated by different lower case letters on top of the figure.

To test for spatial fidelity, we analyzed whether the original position where a worker was collected (near brood, near queen, nest entrance, outside) was a predictor for its whereabouts over the following three weeks. Indeed, workers collected outside were most likely to be found outside, and ones collected near brood stayed near the brood or the queen most of the time. Workers collected at the entrance stayed there significantly more often than those from the other origins, but nevertheless spent most of the time outside the nest. Workers collected near the queen stayed there significantly more often than those from the other origins, but nevertheless spent most of the time near the brood. The linear mixed-effects model showed significant effects of original position for all four origins investigated (all F_3,387_≥7.66, p<0.0001), except for the origins ‘near queen’ and ‘near brood’, which did not differ in any of the three positions scanned (Fig. 1). Hence, we decided to pool queen and brood data for the following analysis as they likely did not represent separate castes. This step is in accordance with the basic caste classification established by Ehrhardt [26] who did not identify a separate queen-tending caste.

### 2. Spatial Flexibility

**Figure 2:**
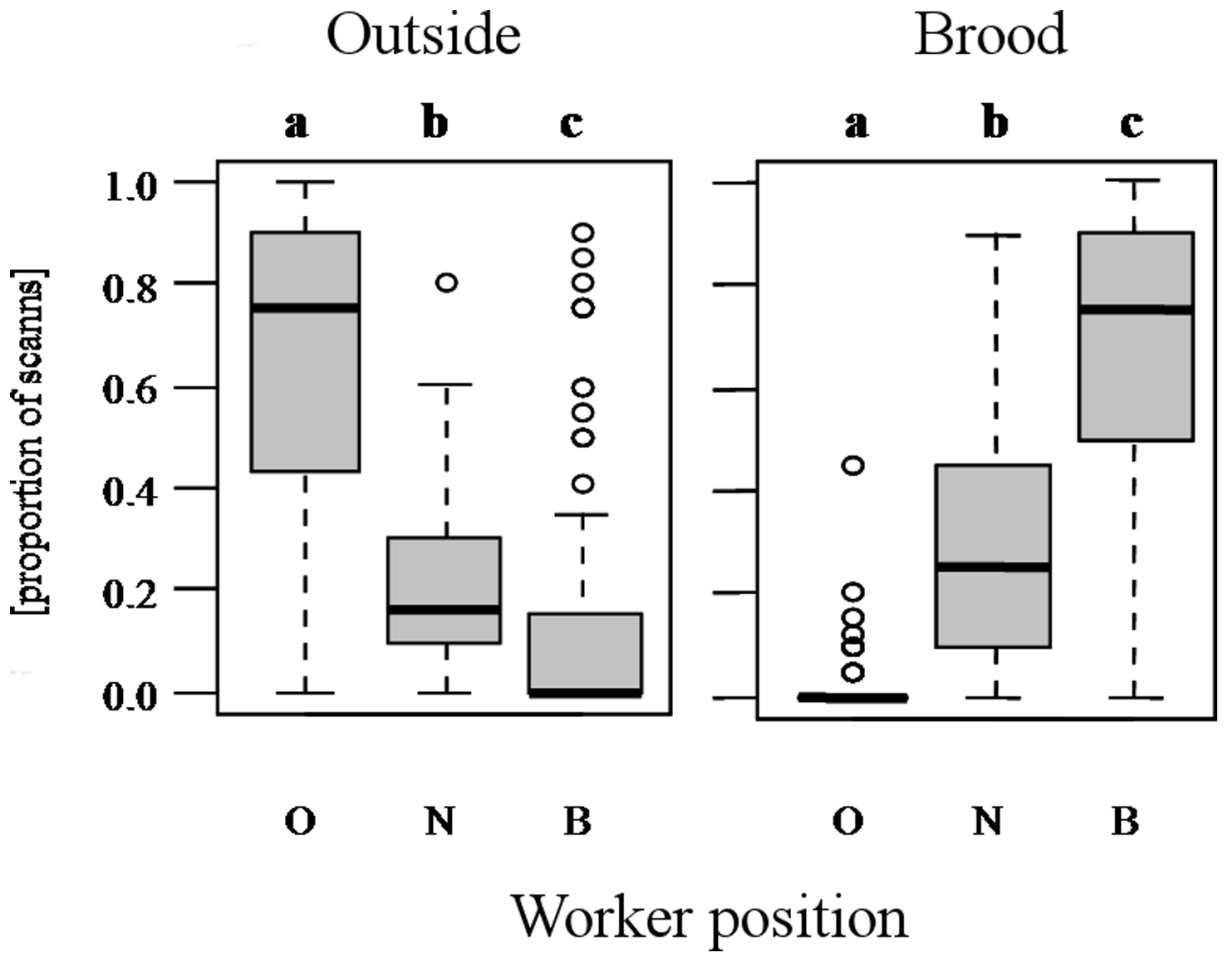
Spatial flexibility of *M. rubra* workers. The graph presents the proportion of scans where an individual was detected at one of the three monitored positions of subnests containing spatial specialists of one type only. We recorded workers present on the outside of the nest (O), within the nest but not at the brood (N) and workers at the brood (B). During this experiment we never detected workers directly in the entrance and consequently decided to monitor workers near the entrance but inside the nest (N). Workers were found more often at the position they were originally located in even after the alteration of colony task demand. Presented are medians and quartiles circles indicate outliers. Significant differences between the positions are indicated by different lower case letters on top of the figure (all p<0.0001).

Following the spatial fidelity experiment, we tested whether the spatially specialized workers from different positions responded to changes in colony task demand by moving to other positions. We set up split nests containing brood and one queen, but only workers from either near the brood or outside the nest. We were interested if workers would switch their position and hence task if all other ants usually performing them were gone. The position the workers were originally found had a significant effect on its spatial position in the split nests during the experiment positions both for brood (F_2,818_ = 104.65, p<0.0001) as well as outside workers (F_2,366_ = 134.86, p<0.0001). Both outside and brood-care workers were found more often at their original position compared to the other two locations (Fig. 2). This indicated that, despite changes in colony workforce demand, workers' current position was influenced by their original position.

### 3. Personality

**Figure 3:**
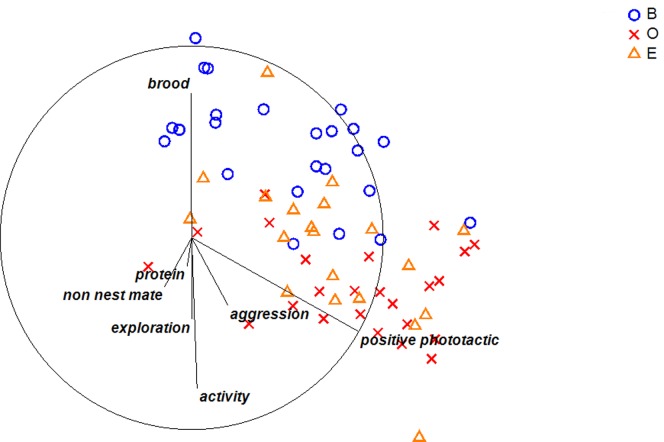
MDS ordination of the worker personality dimensions (2D stress = 0.11), based on euclidian distances. Each symbol represents an individual worker. Lines indicate the contribution of each behavioral trait to the separation among the three groups investigated (B = brood, O = outside and E = entrance). All three groups are significantly different from each other (all p<0.007). The three main contributors to group separation are phototactic (Experiment 3a), activity (3c) and brood-care tendency (3f). The interest in protein foraging (3e), exploration (3b), non-nestmates (3d) and aggression (3g) contributed to a lesser extent.

Following the confirmation of spatial fidelity and the inflexibility of such specialists to relocate, we were interested if these worker groups are characterized by a different “personality”, i.e. by a position-specific position of personality traits. In a first step, we computed a PCA on all behaviors tested (3a–g) in order to reduce the number of behavioral variables. This PCA yielded three principal components with an eigenvalue >1 (Table S1 in File S1). The loadings of the first PC showed strong positive loadings of phototactic behavior, general activity, exploration, aggression and negative loadings of all these behaviors with brood care (Fig. 3, Table S1 in File S1). The values of the first PC differed significantly among the three positions (brood, entrance and outside) (PERMANOVA: Pseudo-F_2,67_ = 170.44, p = 0.0001). No significant effects of colony membership nor interaction between position and colony was found (both F <0.9, p>0.91). The three positional groups all differed significantly (inside vs. entrance: t = 21.6, p = 0.0002; inside vs. outside: t = 15.75, p = 0.0005; entrance vs. outside: t = 6.37, p = 0.007; Fig. S1) with foragers being the most active, positive phototactic, explorative and aggressive workers while brood workers showed the contrary tendency and a higher affinity towards brood (factor loadings for PC1). Workers from the entrance scored intermediate for the first PC (Fig. 3). The second PC showed positive loadings of the interaction time with dead ants and the interaction time with the cricket leg and a negative loadings of aggression (Table S1 in File S1 ). Its values differed significantly among the three positional groups (PERMANOVA: position: Pseudo-F_2,67_ = 15.05, p = 0.006; but not between colonies colony and interaction: both Pseudo-F <1.4, both p>0.22). Entrance workers showed significantly more interest in cricket legs and intruders compared to both inside and outside workers (entrance vs. inside: t = 3.77, p = 0.03; entrance vs. outside t = 4.67, p = 0.015), but inside and outside workers did not differ (t = 1.41, p = 0.26; Fig. S2). The coordinates on the third PC did not differ among the three groups, and there was no effect of colony nor a significant interaction (all p>0.05; Table S1 in File S1).

#### 3.1 Personality persistence

To see if the three focal behavioral traits (activity, phototactic behavior and brood care) were consistent over time, we tested individuals twice with 10 days in between. We chose these three traits because they contributed most to group separation in the personality analysis (Fig. 3). We computed two PCAs: the first PCA (PCA1) on all three behaviors on the first - and a second PCA (PCA2) for all three behaviors on the second day. Both PCAs showed a very similar PC loadings and Eigenvalues for the first two PCs both with an Eigenvalue <1 (regarding: factor loadings, Eigenvalue and per cent variation explained, see Table S2 and S3 in File S1). The first PC showed a positive association between activity and phototactic behavior while the second PC only represented brood care behavior. To test if individuals were consistent in the recorded behaviors we calculated an ANCOVA analysis accounting for the non independence of the workers. The ANCOVA revealed a significant effect of PC1 (second test), indicating a strong correlation between the two PCs (F_1,53_ = 11.41, p = 0.0014) but no effect of colony and no interaction between the two factors (both F_5,53_<0.52, p>0.76). These results indicate that workers showed consistent, context independent behavioral syndromes. The second ANCOVA indicated a significant correlation between PC2 on both testing days (F_1,53_ = 13.82, p = 0.0005), differences between colonies (F_5,53_ = 4.74, p = 0.001) but no interaction between the two factors (F_5,53_ = 1.29, p = 0.28). The results showed that workers exhibit consistent interest in brood but that the workers of different colonies differ in their overall affinity to brood.

### 4. Physiology

Worker behavior might be determined by morphological and physiological parameters. We consequently tested whether workers from different positions showed differences regarding body allometry, reproductive status and cuticular hydrocarbon profile.

#### 4.1 Morphology

In a first step we tested whether there is evidence for worker allometry in *M. rubra* over all workers investigated. A Principal Components Analysis yielded one axis (PC1, Eigenvalue: 3.36) explaining 84% of the variance. The following axes had Eigenvalues of 0.33 or less. All four morphological measures were highly correlated to PC1 (all factor loadings >0.86), and PC1 was normally distributed (Kolmogorov-Smirnov d = 0.085, p = n.s.). Thus, there was no evidence for allometry or separate morphological castes.

#### 4.2 Ovary status

The average ovariole length differed strongly among workers from different positions, but not among colonies (PERMANOVA: position: Pseudo-F_2,54_ = 21.1 p = 0.006; colony and interaction: both Pseudo-F <1.1, p>0.36). Brood workers had significantly longer ovaries than entrance (t = 4.6, p = 0.03) and outside workers (t = 5.28, p = 0.028), but ovary length in entrance and outside workers did not differ (t = 0.47 p = 0.66; see Fig. S3).

#### 4.3 Cuticular hydrocarbon profiles

**Figure 4:**
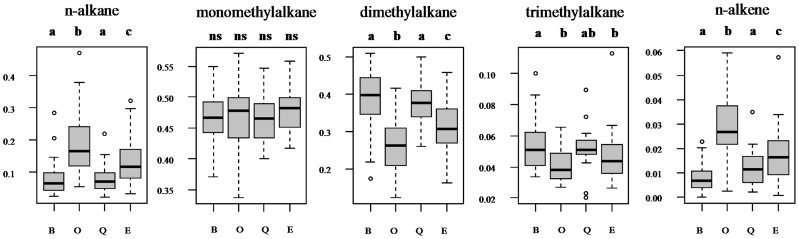
We present the differences between the five analyzed CHC substance classes (left to right). Proportion of the five substance classes. Median and quartiles are presented. Significant differences between the four groups investigated (B = brood, O = outside, Q = queen, E = Entrance) are indicated by the lower-case letters on top of the individual graphs (all significant p<0.003; ns = no significant difference). Circles indicate outliers.

The cuticular hydrocarbon profiles differed significantly between positions in the nest (PERMANOVA: Pseudo-F_3,116_ = 9.182, p = 0.0001) and between colonies (Pseudo-F_15,116_ = 5.49, p = 0.0001). The interaction between position and colony was not significant (Pseudo-F_41,116_ = 1.18, p = 0.11; Fig. S4). Pairwise comparisons revealed significant differences between most groups (outside vs. entrance t = 2.3282, p = 0.0036; outside vs. brood t = 4.61, p = 0.0001; outside vs. queen t = 3.51, p = 0.0005; entrance vs. brood t = 2.85, p = 0.0007), but no difference between entrance- and queen workers (t = 1.7, p = 0.07) nor between brood- and queen workers (t = 1.04, p = 0.39). The relative abundances of *n-*alkanes, *n-*alkenes, and dimethyl and trimethyl alkanes each differed between the positions (LME: all F >8.8, p<0.0001), whereas the relative abundance of monomethyl alkanes did not differ (F = 0.97 p = 0.4; Fig. 4). Outside workers had the highest relative amounts of *n-*alkanes, followed by entrance workers. Queen- and brood tenders had the lowest amounts of *n-*alkanes. A similar pattern was found for *n-*alkenes, but these occurred in far lower abundances than *n-*alkanes. The relative abundances of dimethyl alkanes followed the reverse pattern, being most abundant in queen- and brood tenders, less so in entrance workers and least abundant in outside workers (Fig. 4; for substance identifications see Table S4 in File S1 ).

## Discussion

In this study, we investigated the differences between behavioral castes in the ant *Myrmica rubra*. We analyzed spatial fidelity, spatial flexibility, personality traits, cuticular hydrocarbons, morphology, and ovary length in workers from outside the nest, from the nest entrance, from near the brood, and from near the queen. While we find no clear indication of a separate brood and queen tending caste, both differed strongly from outside and entrance workers (foragers and guards) in respect to all of the above traits (except for morphology). While outside and entrance workers were not separable in their ovary length, they differed in their personality traits, their cuticular hydrocarbons, and, partly, their preferred position in the nest.

How can division of labor emerge from these differences? In the following, we argue that personality differences can lead to differences in spatial position and task preference.

### Personality and Preferred Position

Similar to most soil-nesting ant species, the space where individual *M. rubra* workers operate can be divided into two environments: the dark inside of the nest and the brighter outside. Because all ants start their life and every consecutive day inside the nest, only two parameters are required to reliably define the spatial position of workers: activity and sensitivity to light (i.e., phototactic behavior). We showed that these two behavioral traits exhibit a context- independent correlation, and that the correlation is stable over the investigated time scale. This trait association provides a simple and robust behavioral mechanism to enable the most basic spatial separation of ant workers in inside and outside workers. Active, positively phototactic workers will rather be outside the nest, whereas less active, negatively phototactic individuals will tend to stay inside. We will refer to this correlated set of behavioral traits as position-associated aspects worker of personality. Many ants, including *M. rubra,* have evolved sophisticated trail pheromones organizing division of labor on the outside of the nest. The behavioral mechanism we describe could facilitate the transition of foragers from the inside to the outside where they will encounter trail pheromones which will further direct them to locations of interest.

### Personality and Task

In order to generate division of labor, the position- associated aspects of personality need to be coupled with low thresholds to perform a task located in the defined positions. This is exactly what we found in *M. rubra*. Inside worker personalities (low activity and low attraction to light) were coupled with a low threshold to interact with brood, while high aggression and exploration was associated to traits favoring a position outside the nest (high activity and high attraction to light). Workers in the entrance position (viewed as a intermediate position) were characterized by intermediate position- associated characteristics and an elevated interest in non-nestmates and cricket legs. Consequently, the observed worker personalities, when viewed as position- and task-associated properties, provide a robust mechanism to organize division of labor. These context-independent correlated traits will result in a separation between inside and outside workers with inside workers taking care of the brood while outside workers will explore and defend the nest. The temporally stable set of position- associated traits may account for the notable lack of spatial flexibility when colony demand for specific tasks changed. If ants only infrequently change their position, as indicated by our spatial flexibility experiment, they will probably not perceive task-relevant cues located in different positions and will have a low probability to respond appropriately. We conclude that these specialized workers do not freely switch between positions, and, hence, tasks.

### Underlying Mechanisms for Variation in Personality Traits

Why do individual workers of the same colony show this notable variation in behavioral syndromes? Two, not mutually exclusive, mechanisms have been proposed to influence caste distribution within the colony: temporal polyethism and genetic variation. Temporal polyethism describes a sequence of task transitions over an individual worker's lifespan and has also been shown for *M. rubra* [26]. It could explain the observed variation in worker personalities within a colony [27], and would suggest that worker personalities can change radically during their lifetime. Alternatively, numerous studies have demonstrated the genetic influence on caste determination often associated with different patrilines [28]. *Myrmica rubra* often exhibits pronounced genetic variation between workers, which, however, is likely caused by functional polygyny rather than polyandry [29]. The combination of strong variation in functional queen number and the documented temporal polyethism makes *M. rubra* an interesting study system to investigate the interaction between genetic and temporal factors that shape worker caste composition and their development over time [30]. Both mechanisms likely result in differences on the physiological level (e.g. resting metabolic rate, hormone titer etc.) which could manifest as the animal personalities we detect. It would be very interesting to investigate the interplay between temporal polyethism and genetic variation, its manifestation on the physiological level and their influence on worker personality.

The question why specific behavioral traits form temporally stable behavioral syndromes is an important one linking the observed syndromes to its physiological and ultimately to its genetic basis. It has been suggested that common physiological control mechanism could be responsible for behavioral syndromes if a single mechanism influences or controls two behavioral traits simultaneously [16]. Hormones can be active in numerous places simultaneously and directly influence behavior in many organisms. Hormones are therefore prime candidates for a proximate physiological control mechanism. Judging by the variation in ovary length, changes in the hormonal titer (e.g. vitellogenin, juvenile hormone, or a combination) are likely to accompany the task transition during the life of *M. rubra* workers [(e.g.) 31]. It has been demonstrated in other study systems that hormones can influence both activity and the visual systems directly modulating how the organisms interact with their surroundings [32], [33]. If hormones have a similar effect in insects the involved hormones could control the potentially position associated syndrome (activity-phototactic) we document in *M. rubra*.

### Caste Differences in the Cuticular Hydrocarbon Profiles

Similar to the different states in ovary activation, we showed that entrance workers, foragers, and brood-tenders each had different cuticular hydrocarbon profiles. In contrast, the profiles of brood- and queen tenders did not differ. Foragers, and, to a lesser degree, guards, possessed significantly more *n-*alkanes than inside workers. Task-specific differences in cuticular hydrocarbon profiles have been shown previously, including honeybees and ants [(e.g.) 34,35]. In several of these cases, outside workers had higher proportions of *n-*alkanes, as has been found here as well. Due to the lack of disruptive structural elements, *n-*alkanes tend to aggregate more tightly than other hydrocarbon classes. Thus, they have a substantially higher melting point than methylbranched alkanes or alkenes, which correlates with their waterproofing ability [36], [37]. The significantly higher, but still low abundance of *n-*alkenes in foragers may enhance the width of the solid-liquid transition phase of the cuticular hydrocarbon layer while still maintaining a high overall melting point [37].

We suggest that these differences are caused by either different hormonal levels, individual adaptation to different climatic conditions inside or outside the nest, or a combination of both. Short-term application of Juvenile Hormone III can trigger changes of CHC profiles [38]. Thus, hormonal changes due to differential ovary activation may result in the task-specific CHC profiles. This differentiation may then be further enhanced by individual acclimatisation to the individual's microclimate [35]. Note that this mechanism is independent from whether task specificity is due to genetic differentiation or age polyethism.

While the primary function of cuticular hydrocarbons is to protect the insect body against desiccation [39], task-specific CHC differences can help to organize the division labor. For example, hydrocarbons of patrollers/scouts at the nest entrance can trigger other workers to start foraging, while hydrocarbons of brood tenders do not have this effect [40]. Thus, individual workers can use the hydrocarbon profile of another individual as a cue to assess the other's task. It seems possible that, following a physiological differentiation due to exposure to different microclimates, CHC profiles also function as signals to other workers. Hence, selection may have resulted in mechanisms for a further task-specific divergence of these profiles to further facilitate the organization of division of labor [40].

## Conclusion

In this study we demonstrate how the analysis of animal personality can be applied as a useful tool when studying division of labor [41]. We uncovered position-associated behavioral syndromes, coupled with position-specific task threshold, which could lead to spatially structured division of labor without central control. Our study shows that analyzing the complex interactions between behavior and morphology can provide new insights into complex processes such as division of labor and uncover potentially simple rules governing such complex processes.

## Supporting Information

Figure S1 **Pairwise comparisons of the PERMANOVA on PC1 (see Table S1 for factor loadings in) between the three tested positions (B = brood, E = entrance, O = outside; all p<0.007).** The PERMANOVA indicate that outside workers are more active, positive phototactic, aggressive and explore more compared to workers found at the brood while workers in the entrance score intermediate on this axis. Significant differences are indicated by the lower-case letters on top of the graph. Presented are mean and SE.
(TIF)

Figure S2 **Differences between workers in the three positions (B = brood, E = entrance, O = outside) according to PC2 (see Table S1 for loadings in File S1).** We find that workers in the entrance have an elevated interest in non nest mates and cricket legs (protein) compared to both other groups (both p<0.03) indicating a separate behavioral caste. Significant differences are indicated by the lower-case letters on top of the graph. Presented are mean and SE.
(TIF)

Figure S3 **Average ovariole length of workers found at the three positions (B = brood, E = entrance and O = outside).** Workers inside the nest (brood **B**) had longer ovaries compared to workers in the entrance (**E**) and outside (**O;**) both p<0.03, while outside and entrance worker did not differ. Presented are mean [mm] and SE.
(TIF)

Figure S4 **Chemical differences between the four spatial positions (B = brood, O = outside, E = entrance, Q = queen-tenders).** We find no differences between B and Q and no difference between Q and E but all other combinations differ significantly (all p<0.003). The MDS plot is based on Bray-Curtis similarity as distance estimate. 2D stress = 0.14.
(TIF)

File S1 **Table S1. Table S2. Table S3. Table S4.**
(DOC)

Raw Data S1 (XLS)
