# Supplementary figures and images for: Worker Personality and Its Association with Spatially Structured Division of Labor
Source: PLoS One. 2014 Jan 30;9(1):e79616. doi: 10.1371/journal.pone.0079616 (PMC3907378; doi:10.1371/journal.pone.0079616)

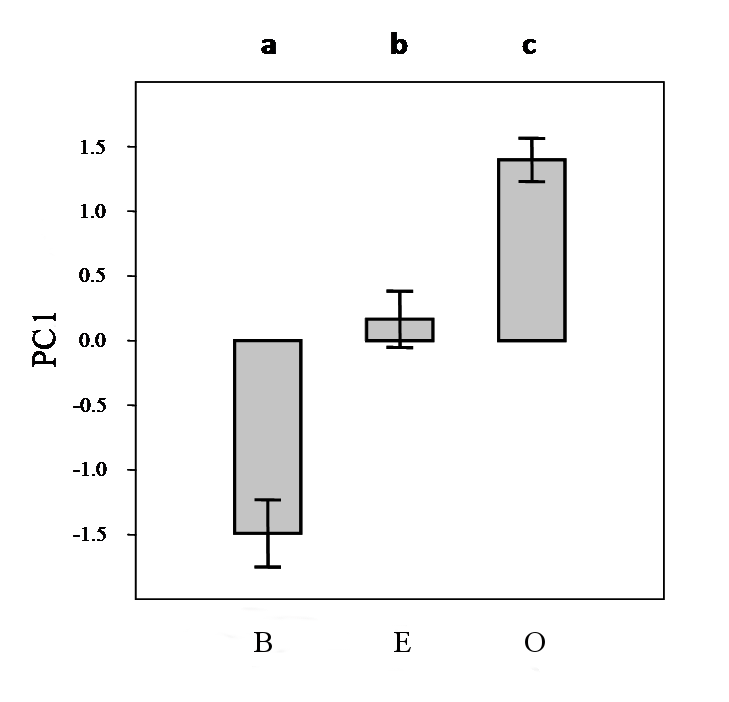

Supplement: Figure S1 — Pairwise comparisons of the PERMANOVA on PC1 (see Table S1 for factor loadings in) between the three tested positions (B = brood, E = entrance, O = outside; all p<0.007). The PERMANOVA indicate that outside workers are more active, positive phototactic, aggressive and explore more compared to workers found at the brood while workers in the entrance score intermediate on this axis. Significant differences are indicated by the lower-case letters on top of the graph. Presented are mean and SE. (TIF) [file pone.0079616.s001.tif]

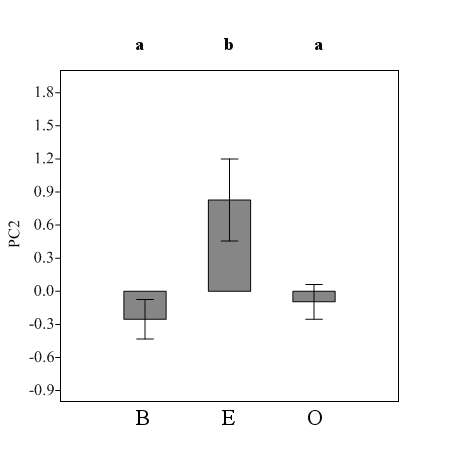

Supplement: Figure S2 — Differences between workers in the three positions (B = brood, E = entrance, O = outside) according to PC2 (see Table S1 for loadings in File S1). We find that workers in the entrance have an elevated interest in non nest mates and cricket legs (protein) compared to both other groups (both p<0.03) indicating a separate behavioral caste. Significant differences are indicated by the lower-case letters on top of the graph. Presented are mean and SE. (TIF) [file pone.0079616.s002.tif]

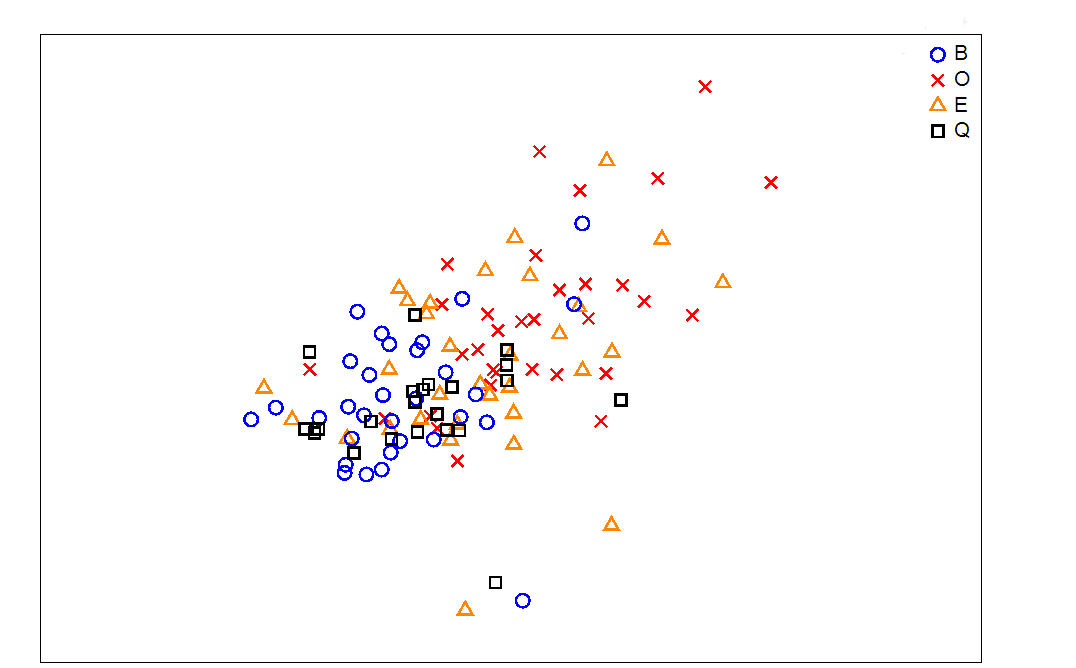

Supplement: Figure S4 — Chemical differences between the four spatial positions (B = brood, O = outside, E = entrance, Q = queen-tenders). We find no differences between B and Q and no difference between Q and E but all other combinations differ significantly (all p<0.003). The MDS plot is based on Bray-Curtis similarity as distance estimate. 2D stress = 0.14. (TIF) [file pone.0079616.s004.tif]
