# Supplementary material for: Worker Personality and Its Association with Spatially Structured Division of Labor
Source: PLoS One. 2014 Jan 30;9(1):e79616. doi: 10.1371/journal.pone.0079616 (PMC3907378; doi:10.1371/journal.pone.0079616)

**Electronic supplemental material**

Worker personality and its association with spatially structured division of labor

Tobias Pamminger, Susanne Foitzik, Katharina Kaufmann, Natalie Schützler and Florian Menzel


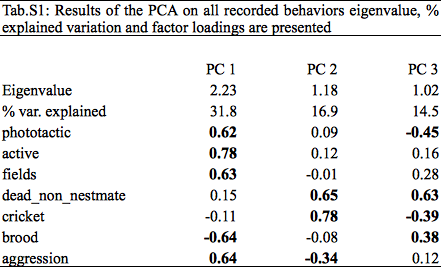


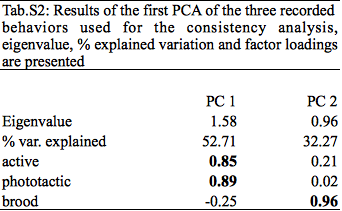


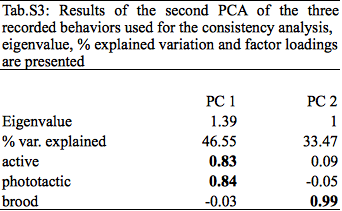


Chemical Analysis:

For each colony and location, two workers were killed by freezing and then immersed into approximately 100µl hexane for 10 min. The extracts were concentrated under nitrogen flow. Five µl were injected in splitless mode into an Agilent 7890A gas chromatograph coupled to a 5975 mass-selective detector (Agilent Technologies), which was equipped with a capillary column HP5-MS (Agilent; 30 m x 0,25mm; df = 0.25µm). The inlet had a temperature of 250°C; helium was used as carrier gas with a flow of 1.2 ml / min. The temperature was kept at 60°C for 2min, then raised by 60 K / min to 200°C and subsequently by 4 K / min to 320°C, where it remained constant for 10 min. The electron impact–mass spectra were recorded with an ionisation voltage of 70 eV, a source temperature of 230°C, and an interface temperature of 320°C. Relative substance quantities were recorded based on total ion counts (TIC) from scans between 40 and 550 amu.


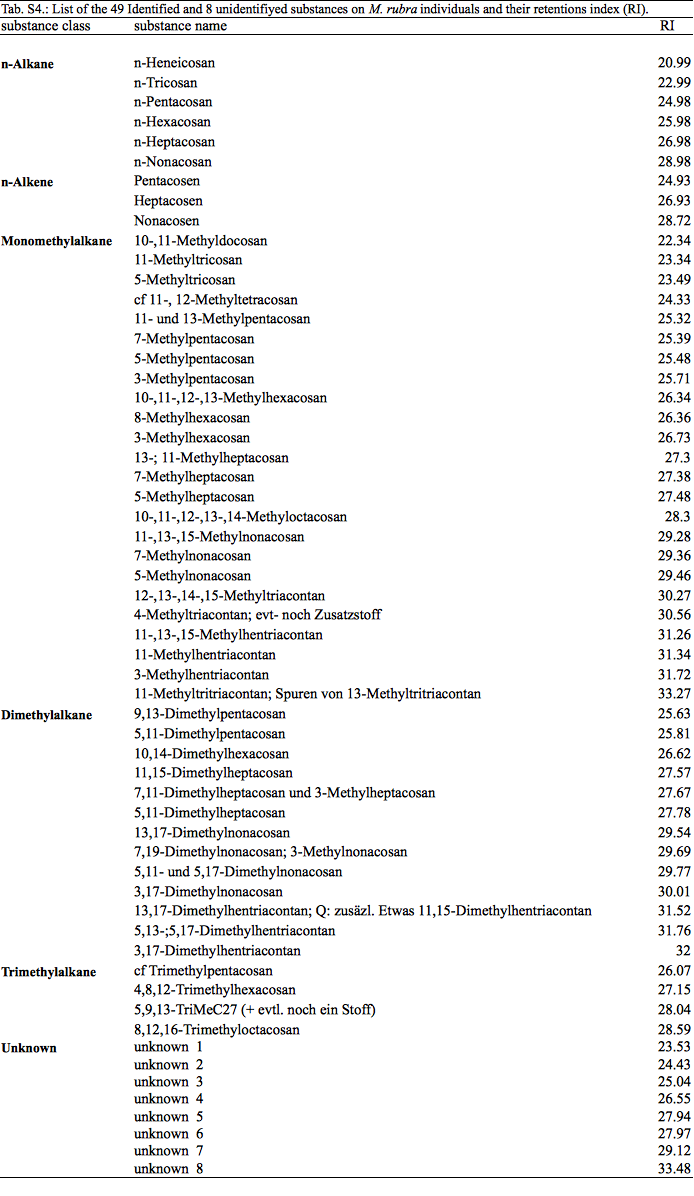

Supplement: File S1 — Table S1. Table S2. Table S3. Table S4. (DOC) [file pone.0079616.s005.doc]
